# Robot-Assisted Radical Prostatectomy for Potential Cancer Control in Patients with Metastatic Prostate Cancer

**DOI:** 10.3390/curroncol29040233

**Published:** 2022-04-18

**Authors:** Kimiaki Takagi, Makoto Kawase, Daiki Kato, Kota Kawase, Manabu Takai, Koji Iinuma, Keita Nakane, Noriyasu Hagiwara, Toru Yamada, Masayuki Tomioka, Takuya Koie

**Affiliations:** 1 Department of Urology, Daiyukai Daiichi Hospital, Ichinomiya 4918551, Japan; kimiaki_takagi5619@yahoo.co.jp; 2 Department of Urology, Graduate School of Medicine, Gifu University, Gifu 5011194, Japan; buki2121@gifu-u.ac.jp (M.K.); andreas7@gifu-u.ac.jp (D.K.); stnf55@gmail.com (K.K.); takai_mb@gifu-u.ac.jp (M.T.); kiinuma@gifu-u.ac.jp (K.I.); keiaco@gifu-u.ac.jp (K.N.); 3 Department of Urology, Matsunami General Hospital, Gifu 5016062, Japan; hagwara44@gmail.com; 4 Department of Urology, Tokai Central Hospital, Kakamigahara 5048601, Japan; toru.yamada@tokaihp.jp; 5 Department of Urology, Japanese Red Cross Takayama Hospital, Takayama 5068550, Japan; tomiokam@gifu-u.ac.jp

**Keywords:** robot-assisted radical prostatectomy, metastatic prostate cancer, neoadjuvant therapy, treatment-free survival, biochemical recurrence

## Abstract

Recently, cytoreductive prostatectomy for metastatic prostate cancer (mPCa) has been associated with improved oncological outcomes. This study was aimed at evaluating whether robot-assisted radical prostatectomy (RARP) as a form of cytoreductive prostatectomy can improve oncological outcomes in patients with mPCa. We conducted a retrospective study of twelve patients with mPCa who had undergone neoadjuvant therapy followed by RARP. The endpoints were biochemical recurrence-free survival, treatment-free survival, and de novo metastasis-free survival. At the end of the follow-up period, none of the enrolled patients had died from PCa. The 1- and 2-year biochemical recurrence-free survival rates were 83.3% and 66.7%, respectively, and treatment-free survival rates were 75.0% and 56.3%, respectively. One patient developed de novo bone metastases 6.4 months postoperatively, and castration-resistant prostate cancer 8.9 months postoperatively. After RARP, the median duration of recovery of urinary continence was 5.2 months. One patient had severe incontinence (>2 pads/day) 24 months postoperatively. RARP may be a treatment option in patients with mPCa who have achieved a serum prostate-specific antigen level < 0.2 ng/mL, and present without new lesions on imaging.

## 1. Introduction

Radical prostatectomy (RP), particularly robot-assisted RP (RARP), is a standard treatment option for patients with clinically localized or locally advanced prostate cancer (PCa) [1,2]. Guidelines recommend androgen deprivation therapy (ADT) with or without chemotherapy for patients with metastatic PCa (mPCa) [3]. However, in a previous study, patients who received ADT alone as the initial treatment had a median time to progression and an overall survival (OS) of 11 and 42 months, respectively [4].

Recently, cytoreductive prostatectomy (CRP) has been associated with improved oncological outcomes in mPCa through large cohort studies using the Surveillance Epidemiology and End Results (SEER) database [5,6]. In addition, CRP has shown a significantly higher survival benefit compared to radiation therapy [5,6]. In a randomized double-blind phase III trial (the Southwest Oncology Group (SWOG) 8894 trial), patients with mPCa who previously underwent RP had a lower risk of cancer-related mortality than those who did not (hazard ratio = 0.77; 95% confidence interval (CI): 0.53–0.89) [7]. Although the role of definitive therapies remains unclear in patients with mPCa, localized therapy could reduce the risk of metastasis by minimizing tumor seeding and changing the tumor microenvironment [8]. Therefore, resection of the primary tumor with a continuous potential to metastasize, including promotion of growth factors and immunosuppressive cytokines, may have advantages of CRP, even though the pathogenetic mechanisms involved in the advantageous effects of CRP are unclear [9].

This study was aimed at evaluating whether or not RARP as a form of CRP can improve oncological outcomes in patients with mPCa.

## 2. Materials and Methods

### 2.1. Patient Population

We retrospectively enrolled twelve patients with mPCa who had undergone neoadjuvant therapy followed by RARP at Gifu University and Matsunami General Hospital between July 2017 and January 2021. We collected preoperative patient characteristics, including the age, height, weight, serum prostate-specific antigen (PSA) and testosterone level, clinical stage, biopsy Gleason score, Eastern Cooperative Oncology Group performance status [10], and type of neoadjuvant therapy. Further, we collected pathological characteristics, including the T and N stages of the surgical specimens, pathological Gleason score, and surgical margins. Tumor staging was performed according to the American Joint Committee on Cancer eighth edition cancer staging manual [11]. RARP-related perioperative complications were evaluated according to the Clavien–Dindo classification [12].

The enrolled patients had received neoadjuvant therapy before RARP. The patients received combination therapy including luteinizing hormone-releasing hormone agonists or antagonists (LHRH) and bicalutamide 80 mg/day as combined androgen blockade therapy, LHRH plus abiraterone 1000 mg/day or apalutamide 240 mg/day as androgen receptor pathway inhibitor therapy, and LHRH and tegafur-uracil 300 mg/day as chemohormonal therapy. We confirmed that they had achieved a serum PSA level < 0.2 ng/mL, maintained a castration level (<50 pg/mL), and presented without new lesions on computed tomography and bone scintigraphy, indicating RARP. Pelvic lymphadenectomy or a nerve-sparing approach had not been used because of distant metastasis at the initial diagnosis.

This study was approved by the Institutional Review Board of Gifu University (approval number: 2019-267) and institutional review boards of the participating institutions. The requirement for informed consent was waived because of the retrospective design. Based on the provisions of the ethics committee and ethical guidelines in Japan, written consent was not required, since the results of the retrospective study using existing documentation had already been disclosed to the public. The details of the study can be found at http://www.med.gifu-u.ac.jp/file/2020-271.pdf (accessed on 20 March 2022).

### 2.2. Histopathology

We evaluated RARP specimens using the whole-mount staining technique and 2014 International Society of Urologic Pathology guidelines [13]. The apical section of the prostate was truncated perpendicular to the prostatic urethra. The bladder neck margin was coned from the specimen, and sectioned perpendicular to it. The remaining prostate tissue was completely sectioned at 3-mm intervals along a plane perpendicular to the urethral axis.

### 2.3. Follow-Up

Following RARP, all patients were assessed at 3-month intervals for serum PSA and testosterone levels. The date of disease recurrence or PSA failure was determined as two consecutive PSA values that exceeded 0.2 ng/mL. When the PSA level did not decrease below 0.2 ng/mL postoperatively, the date of RARP was defined as the date of PCa recurrence.

### 2.4. Endpoints and Statistical Analysis

The endpoints were biochemical recurrence-free survival (BRFS), treatment-free sur vival (TFS), and de novo metastasis-free survival (dMFS). De novo metastasis was defined as a new lesion after neoadjuvant therapy followed by CRP. Data were analyzed using SPSS version 24.0 (IBM Corp., Armonk, NY, USA). BRFS, TFS, and dMFS after RARP were analyzed using the Kaplan–Meier method. dMFS was defined as the time from RARP to the appearance of de novo locoregional and/or distant metastasis on computed tomography and/or bone scintigraphy. Two-sided *p*-values < 0.05 were considered to be statistically significant.

## 3. Results

### 3.1. Patient Characteristics

Table 1 presents the patient demographic data. Twelve patients were enrolled in the study. The highest initial serum PSA level was 1623.229 ng/mL. All patients presented with bone metastasis at the initial PCa diagnosis. Two patients underwent combined ADT for over 100 months before RARP. The median duration of administration of androgen receptor pathway inhibitors or chemohormonal therapy before RARP was 6.3 months. None of the enrolled patients had severe urinary tract symptoms before initiating neoadjuvant therapy or CRP. The enrolled patients achieved primary site reduction and complete remission of lymph node metastasis after neoadjuvant therapy. Five patients had bone metastasis without new lesions on bone scintigraphy before CRP.

### 3.2. Surgical and Pathological Outcomes

Table 2 shows the surgical and pathological outcomes. None of the enrolled patients developed RARP-related complications. The console time was relatively shorter, whereas the estimated blood loss was relatively lower compared to that in previous studies [14,15,16].

The median duration of urinary continence was 5.2 (0.6–8.9) months. One patient developed severe incontinence (>2 pads/day) 24 months postoperatively.

### 3.3. Oncological Outcomes

At the end of the follow-up period, none of the enrolled patients had died from PCa. One patient died from other causes (details unknown). Biochemical recurrence after RARP was identified in three patients (two patients on the day of RARP, and one patient 14.1 months later).

**Figure 1:**
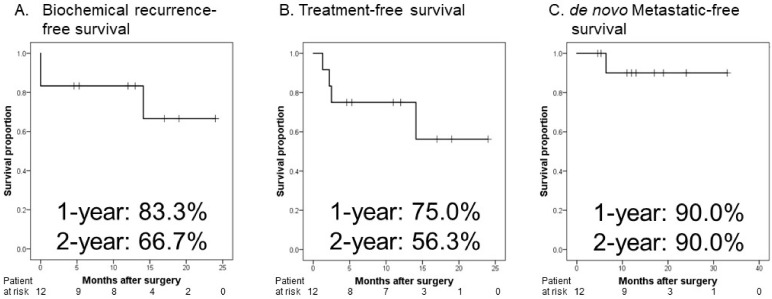
Kaplan–Meier estimates of oncological outcomes in patients with metastatic prostate cancer who received neoadjuvant therapy followed by robot-assisted radical prostatectomy. (**A**) The 1-and 2-year biochemical recurrence-free survival rates are 83.3% and 66.7%, respectively. (**B**) The 1-and 2-year treatment-free survival rates are 75.0% and 56.3%, respectively. (**C**) The 1- and 2-year de novo metastatic-free survival rates are 90.0% and 90.0%, respectively.

Figure 1 shows the Kaplan–Meier curves. The 1- and 2-year BRFS rates were 83.3% and 66.7%, respectively (Figure 1A), and TFS rates were 75.0% and 56.3%, respectively (Figure 1B). Regarding dMFS, one patient developed de novo bone metastases 6.4 months postoperatively, and castration-resistant PCa 8.9 months postoperatively (Figure 1C).

## 4. Discussion

ADT is the recommended treatment modality for patients with mPCa [3]. However, more than one-third of patients with mPCa who do not receive local treatment for the primary lesion experience significant urinary tract complications because of local progression of PCa [17]. In addition, mPCa comprises a heterogeneous population of patients with distinct prognoses between 11 and 75 months [4,18]. The prospective randomized SWOG 9346 trial, involving 1078 patients with mPCa, was stratified based on the amount of PSA decrease with a significantly different median survival of 13 months for PSA > 4 ng/mL, 44 months for PSA > 0.2 ng/mL, and 75 months for PSA < 0.2 ng/mL [18].

Recently, several retrospective studies have reported oncological outcomes in patients with mPCa who underwent RP [5,6,19]. In 2014, Culp et al. evaluated patients with stage IV PCa who underwent RP, brachytherapy, and no surgery or radiation in the SEER database [5]. A total of 8185 patients, including 245 who underwent RP, were enrolled in this study [5]. The 5-year OS and cancer-specific survival rates in patients with M1a/b/c who underwent RP were significantly higher than those who received no surgery or radiation [5]. To reduce the effects of selection bias and potential confounders in this observational study, a propensity score analysis was performed on the SEER–Medicare linked database [6] and National Cancer Database [19]. Satkunasivam et al. reported a 45% lower risk of cancer-specific mortality in patients who underwent RP than in those who did not receive local treatment after propensity-score-matching using several covariates, including the age, PSA level, tumor stage or grade, comorbidity index, or use of ADT [6]. Similarly, the 3-year OS was 66% in patients who underwent local treatment, and 51% in those who did not after adjusting for the age, PSA level, tumor grade, tumor/node/metastasis classification, and comorbidity index [19]. However, only 20% of the patients in this series underwent local treatment [19].

Although the role of localized therapies in mPCa remains unclear, several hypotheses have been proposed. Kim et al. reported that the primary tumor can act as the source of circulating tumor cells with the potential of “self-seeding” of the primary tumor [20]. Another hypothesis is based on the seed and soil theories [21]. The primary tumors metastasize by disseminating tumor cells into the circulation, and preparing the so-called “premetastatic niche” for metastasis implantation [21]. The proliferation of metastasis at distant sites is stimulated and maintained by compounds secreted by the primary tumor into the circulation [22]. Based on these theories, localized treatment of the prostate in patients with mPCa may inhibit the initiation of distant metastases and progression of existing metastatic sites [23]. Thus, the removal of androgen-intensive clones from the primary tumor may explain why patients after CRP have a longer time to ADT failure, and a better response to ADT [7,13].

CRP may have potential advantages in terms of oncological outcomes. However, CRP may have addressed several surgery-related complications. Preissler et al. compared the surgical outcomes of RP between patients with metastatic PCa (*n =* 953) and those with localized PCa (*n =* 75,425) [24]. CRP in patients with mPCa had a significantly higher rate for overall, intraoperative, genitourinary, and miscellaneous complications and blood transfusion compared to that in patients with localized PCa [24]. In the Local Treatment of Metastatic Prostate Cancer (LoMP) trial, the median operative time was 215 min, and median blood loss volume was 250 mL [13]. Within 3 months after RP, five (29.4%) and two (11.8%) patients developed grade 1 and 2 complications, respectively [12].

The current study had several limitations. First, this was a retrospective study with an inherent potential for bias. Second, the study had a strong vital weakness regarding the oncological advantages of CRP because a relatively small number of patients were enrolled, and the follow-up period was relatively short. Therefore, we reported no functional or long-term oncological outcomes. Additionally, we could not perform multivariate analysis to determine the predictive factor according to the improvement of oncological outcomes in patients with mPCa who received neoadjuvant therapy followed by CRP. Third, no control group of patients received radiation therapy or ADT alone for mPCa. Therefore, the CRP-related morbidity should be informed to the patient, and shared decision-making between the patient and physician after discussing the possible advantages and disadvantages should be encouraged. Finally, there was no standardized systemic treatment protocol before and after CRP. The conclusions are hypothesis-generating. Careful observation of the oncological and functional consequences for a long period is necessary.

## 5. Conclusions

Patients with mPCa who underwent neoadjuvant therapy followed by RARP achieved favorable oncological outcomes, particularly de novo metastasis-free survival, although the follow-up period was relatively short. In addition, more than half of the patients did not receive further treatment for mPCa after RARP. RARP may be a treatment option in patients with mPCa who have achieved a serum PSA level <0.2 ng/mL, and present without new lesions on computed tomography and bone scintigraphy.

## Figures and Tables

**Table 1:** Patient characteristics.

Variables	
Age (year, median, IQR)	74.5 (62.0–78.8)
Body mass index (kg/m^2^, median, IQR)	23.8 (22.5–25.0)
Initial PSA (ng/mL, median, IQR)	185.300 (34.944–333.379)
Clinical T stage (number, %)	
Biopsy Gleason score (%)	
4 + 3	2 (16.7)
4 + 4	2 (16.7)
4 + 5	6 (49.9)
5 + 4	2 (16.7)
Clinical T stage (number, %)	
T2	5 (41.7)
T3	5 (41.7)
T4	2 (16.6)
Clinical N stage (number, %)	
N0	5 (41.7)
N1	7 (58.3)
Clinical M stage (number, %)	
M0	0
M1	12 (100)
Metastatic sites (number, %)	
Lymph nodes	7 (58.3)
Bone	12 (100)
Number of bone metastasis (%)	
1	5 (41.7)
3	5 (41.7)
≥5	2 (16.6)
Neoadjuvant therapy (number, 5)	
Combined androgen blockade	4 (33.3)
ARPI	3 (25.0)
Chemohormonal therapy	5 (41.7)
Duration of neoadjuvant therapy (months, median, IQR)	7.3 (6.1–27.2)

IQR: interquartile range; PSA: prostate-specific antigen; ARPI: androgen receptor pathway inhibitor.

**Table 2:** Surgical and pathological outcomes.

Variables	
Proximate PSA before RARP (ng/mL, median, IQR)	0.017 (0.007–0.194)
Console time (min, median, IQR)	85.0 (70.3–112.0)
EBL (mL, median, IQR)	23 (7–45)
pathological T stage (number, %)	
T0	5 (41.7)
T2	5 (41.7)
T3	2 (16.7)
Pathological Gleason score (%)	
0	5 (41.7)
3 + 4	1 (8.3)
4 + 3	2 (16.7)
5 + 3	1 (8.3)
Positive surgical margin (number, %)	1 (8.3)
Adjuvant ADT (number, %)	3 (25.0)

PSA: prostate-specific antigen; RARP: robot-assisted radical prostatectomy; IQR: interquartile range; EBL: estimated blood loss; ADT: androgen deprivation therapy.

## Data Availability

The data presented in this study are available on request from the corresponding author. The data are not publicly available due to privacy and ethical reasons.
